# Pediatric Thyroid Cancer: To Whom Do You Send the Referral?

**DOI:** 10.3390/cancers13174416

**Published:** 2021-09-01

**Authors:** Allison Keane, Darrin V. Bann, Meghan N. Wilson, David Goldenberg

**Affiliations:** Milton S. Hershey Medical Center, Department of Otolaryngology-Head and Neck Surgery, Penn State Health, 500 University Drive, Hershey, PA 17033, USA; akeane@pennstatehealth.psu.edu (A.K.); darrin.bann@unchealth.unc.edu (D.V.B.); mwilson18@pennstatehealth.psu.edu (M.N.W.)

**Keywords:** pediatric thyroidectomy, surgeon volume, surgeon subspecialty, multidisciplinary

## Abstract

**Simple Summary:**

Pediatric thyroidectomies are performed by pediatric and adult general surgeons as well as pediatric and adult otolaryngologists. Surgeons may be high or low-volume thyroid surgeons. In this review, we discuss the roles of surgical subspecialty, surgeon volume, and institution volume as they relate to pediatric thyroidectomy outcomes. We also present institutional approaches to multidisciplinary treatment of pediatric thyroid cancer.

**Abstract:**

Pediatric thyroid cancer is rare, but increasing in annual incidence. Differentiated thyroid cancer in pediatric patients is treated surgically. Pediatric thyroidectomies are performed by general surgeons, otolaryngologists, general pediatric surgeons, and pediatric otolaryngologists. In a comprehensive literature review, we discuss the evidence supporting the importance of surgeon subspecialty and surgeon volume on outcomes for pediatric thyroid cancer patients. Pediatric general surgeons and pediatric otolaryngologists perform most pediatric thyroidectomies. Certain subpopulations specifically benefit from a combined approach of a pediatric surgeon and a high-volume thyroid surgeon. The correlation between high-volume surgeons and lower complication rates in adult thyroid surgery applies to the pediatric population; however, the definition of high-volume for pediatric thyroidectomies requires further investigation. The development of dedicated pediatric thyroid malignancy centers and multidisciplinary or dual-surgeon approaches are advantageous.

## 1. Introduction

Pediatric thyroid cancer is rare, with an annual incidence of 11.4 cases per 1,000,000 children [1]. However, it is the fourth most common overall malignancy in children, the most common endocrine malignancy, and the incidence is increasing annually [1,2]. The incidence of pediatric thyroid cancer varies by age. Pediatric is defined differently in studies, either as patients under 18 years old or 19 years old. Patients 15 to 19 years old have the highest incidence of thyroid cancer, and children under age 5 have the second-highest incidence [1,2]. Caucasian females 13 to 14 years old are the most commonly affected population [2,3,4,5,6]. Females are affected four times more often than males, although the gender ratio varies with age at diagnosis [2]. 

Risk factors for pediatric thyroid cancer include radiation exposure, iodine insufficiency, and genetic syndromes associated with thyroid malignancy. Pediatric malignancies treated with radiation place a patient at higher risk of developing a secondary thyroid malignancy later in life. The risk of developing a thyroid malignancy secondary to radiation exposure is correlated with the amount of exposure and age at initial exposure. Iodine deficiency, a risk factor for thyroid disease in the adult population, is similarly a risk factor in the pediatric population. Multiple genetic syndromes are associated with thyroid malignancy. Familial adenomatous polyposis (FAP) results from a genetic defect in the APC gene. Colonic polyps and papillary thyroid cancer are characteristic of FAP. Carney complex, DICER1 syndrome, and PTEN hamartoma syndrome are associated with multinodular goiters, follicular adenomas, and differentiated thyroid cancer. Multiple endocrine neoplasia (MEN) type 2A and 2B are associated with medullary thyroid cancer. MEN syndrome and medullary thyroid cancer can be sporadic or inherited. 

Papillary, follicular, papillary follicular variant, and medullary thyroid cancer are the most frequent subtypes encountered in pediatric patients. Papillary subtype accounts for the majority of cases [2]. The incidence of the papillary subtype increases with patient age, and for children aged five through nineteen, papillary subtype is most common [2]. The incidence of medullary subtype decreases with patient age. In children under five years of age presenting with thyroid cancer, medullary thyroid cancer has the highest incidence [2].

When a pediatric patient presents with a thyroid nodule, there is a 2–5-fold increased risk of malignancy compared to adults [3]. Approximately 25% of thyroid nodules in children are determined to be malignant [4,7,8]. The 2015 American Thyroid Association (ATA) guidelines recommend ultrasound evaluation of all pediatric thyroid nodules [9]. Nodule size is not an indication of fine needle aspiration. The decision to biopsy is recommended based on the presence of concerning ultrasound features such as irregular margins, hypoechogenicity, nodule vascularity, microcalcifications, and lymphadenopathy [9]. The Bethesda classification is used to determine the risk of malignancy in pediatric thyroid nodules, as it is with adults. However, studies have shown the risk of malignancy for each classification may be higher for pediatric patients [10,11].

Pediatric patients with Bethesda I or II biopsy classification are recommended for observation and repeat ultrasound and/or fine-needle aspiration in 3–6 or 6–12 months [9]. The ATA recommends thyroid lobectomy and isthmusectomy for pediatric patients meeting Bethesda classification III or IV, and total thyroidectomy is recommended for patients with classification V or VI [9]. Indications for a central neck dissection are less clear. The 2015 ATA guidelines recommend considering a central neck dissection at the initial surgery because data has shown disease-free survival correlates with persistent or recurrent locoregional disease [9,12,13,14]. Lateral neck dissection is recommended only for confirmed metastatic disease and not in a prophylactic manner [9,12,13,14]. 

Compared to adults, pediatric thyroid cancer more often presents with lymph node involvement and metastases [3]. Despite more advanced disease at presentation, pediatric patients have a higher disease-free survival rate than adults. Survival rates for pediatric patients vary by the age of onset and metastases. Patients diagnosed below age ten have a significantly higher 15-year disease-specific survival rate than pediatric patients diagnosed age ten or above, but the 15-year disease-specific survival remains greater than 95% in both groups [2]. Metastatic disease at diagnosis is a risk factor for decreased survival rate in pediatric patients regardless of cancer subtype; however, this is most apparent with medullary subtype [2]. Dermody et al. found a 15-year disease-specific survival rate of 50% for pediatric medullary thyroid cancer with metastases at presentation, compared to about 75% for those with no metastases at presentation [2]. 

For malignant or benign disease, complications related to thyroid surgery are higher in the pediatric population when compared to adults [15]. Complications are also more common in children under six years old, with the highest complication rates reported in children under one year old [16]. The most common complication after a pediatric thyroidectomy is hypocalcemia, occurring at rates of 13–20% [16,17]. Risk factors for hypocalcemia in the pediatric population include malignant disease, Graves’ disease, total thyroidectomies, and lymph node dissections [16,17]. Additional, less-frequent complications include, but are not limited to, unilateral permanent or transient vocal cord immobility, bilateral permanent or transient vocal cord immobility, wound dehiscence, surgical site infection, neck hematoma, or prolonged hospital stay (Table 1). Vocal cord paralysis is reported at 1.8% in total thyroidectomy and 1.5% in partial thyroidectomy [16]. Overall, complications are correlated with prolonged length of stay and increased hospital costs [16]. Complications after adult thyroid surgery are associated with surgeon experience, measured by case volume [18,19]. The higher rate of complications in pediatric thyroidectomies has also been hypothesized to be attributed to surgeon experience, especially given the relative rarity of the disease [3,6]. However, there is conflicting evidence to support this hypothesis, and the definition of a high-volume pediatric thyroid surgeon remains unclear.

Given the relative rarity of pediatric thyroid cancer, increased risk of complications, and the importance of appropriate surgical treatment on disease-free survival, the question arises as to who should be performing pediatric thyroid surgery. Pediatric thyroidectomies are typically performed by general surgeons, pediatric surgeons, otolaryngologists, and pediatric otolaryngologists. Specific skillsets, techniques, and training vary between the specialties, all of which have aspects and nuances applied to pediatric thyroidectomies. There are conflicting reports about differences in complication rates based on surgical subspecialty. Studies have compared both pediatric and non-pediatric surgeons and general and otolaryngologic surgeons in addition to high-volume and low-volume surgeons. This review discusses the evidence supporting the importance of surgeon subspecialty and surgeon volume on outcomes for pediatric thyroid cancer patients. We further discuss the development of pediatric thyroid malignancy centers and multidisciplinary approaches to the care of this patient population. 

## 2. Impact of Surgeon Subspecialty 

Pediatric-trained surgeons commonly operate on pediatric thyroid disease, reportedly performing 75% of the thyroidectomies in recent studies [5,20]. Table 2 compares the reported pediatric thyroid surgery rates among different surgical subspecialties. The National Surgical Quality Improvement Program (NSQIP-P) data from 2015–2017 reports 18% of pediatric thyroidectomies are by non-pediatric general surgeons and 7% by non-pediatric otolaryngologists [5]. Pediatric general surgeons performed most thyroidectomies (42%); this is followed by pediatric otolaryngologists (33%) [5]. Comparison with other recent studies shows general pediatric surgeons consistently perform most pediatric thyroid surgeries (Table 2). From a demographic perspective, in recent studies, non-pediatric surgeons are more likely to operate on adolescent patients and Caucasian patients [5]. Prior studies, such as Tuggle et al., report a higher incidence of pediatric thyroidectomies performed by non-pediatric surgeons [8]. This is consistent with the evolution of pediatric surgery and pediatric otolaryngology as subspecialty fields. 

Utria et al. found that non-pediatric surgeons are involved more often in thyroidectomies for malignant pathology, particularly advanced malignant disease requiring a neck dissection [5]. Of the thyroidectomies performed by non-pediatric surgeons, 36% were for malignant pathology, compared to 27% of thyroidectomies performed by pediatric surgeons [5]. Seventy percent of the patients with malignant pathology had thyroidectomy performed by a pediatric surgeon, but only 58% of patients requiring neck dissection had surgery by a pediatric surgeon [5]. In their analysis comparing only pediatric-trained surgeons, Drews et al. also found that pediatric otolaryngologists more commonly performed surgeries for a malignancy, MEN, or thyroid nodules [21]. Others have reported similar findings. From the Healthcare Cost and Utilization Project Nationwide Inpatient Sample, Tuggle et al. reported that 38% of the cases operated on by otolaryngologists involved malignant disease, compared to 25% of the general surgery cases [8]. Savoca et al. similarly report that 49% of pediatric otolaryngology thyroid procedures were for benign neoplasms and 28% for malignant neoplasms, compared to 35% and 21% of general pediatric surgeons [20]. Data regarding the frequency of procedure performed, partial thyroidectomy, or total thyroidectomy, varied within studies. Drews et al. report that pediatric otolaryngologists are more likely to perform total thyroidectomies [21]. However, this was inconsistent with the data presented by Savoca et al [20]. Despite this, pediatric otolaryngologists and non-pediatric surgeons were consistently more likely to perform a neck dissection [5,8,20,21]. Patients with malignant disease and those that require a neck dissection are more likely to be operated on by a pediatric otolaryngologist or a non-pediatric physician. 

Complications related to thyroid surgery are listed in Table 1. Outcomes postoperatively were compared between pediatric and non-pediatric surgeons, and complications were low and similar [3,5]. Utria et al. reported readmissions as postoperative complications and found similar rates among pediatric and non-pediatric surgeons, 3% and 2%, respectively. Al-Qurayshi et al. defined complications as surgical or medical complications related to thyroidectomy. The most common complications were hypocalcemia and vocal fold immobility. The risk of postoperative complications did not vary by pediatric and non-pediatric surgeons [3]. The general complication rate, nerve injury rate, and length of stay are higher in children under six years of age [15]. This increased complication rate is attributed to age and the increased rate of medullary cancer in this age group, not surgeon specialty. Adult surgeons are more likely to operate on adolescent patients, while pediatric surgeons more commonly treat younger patients [8]. Despite this, and the known increased complication risk in patients under six years old, the complication rates do not vary between pediatric and non-pediatric surgeons. Pediatric surgeons and pediatric otolaryngologists are specifically trained in pediatric anatomy. They have high comfort and competence with small operative fields, making them well equipped for pediatric thyroidectomies in the youngest patients. 

Outcomes of pediatric general surgeons and pediatric otolaryngologists have been directly compared. Drews et al. found that patients treated by pediatric surgeons had a complication rate of 14%, compared to 22.5% in patients treated by pediatric otolaryngologists [21]. When evaluating patients with malignancy, pediatric surgeons had a complication incidence of 25%, compared to pediatric otolaryngologists with a 42% incidence [21]. Patients with benign disease also had a higher incidence of complications with pediatric otolaryngologists [21]. This is contrary to other studies that revealed differences in outcomes based on surgeon volume but did not find statistically significant differences in outcomes or complications based on surgeon subspecialty [3,8]. Al-Qurayshi et al. found no difference in medical and surgical complications between pediatric, otolaryngology, and general surgeons [3]. Savoca et al. reviewed NSQIP-P data from 2012 through 2016 and directly compared pediatric otolaryngologists and pediatric general surgeons. They did not find a difference in adverse outcomes or complications between the two groups [20].

Comparing complication rates by surgical subspecialty is difficult, given the disparity in what is encompassed in the complications. The complications reported by Utria et al. include only those requiring readmission and may not have included all hypocalcemia events or nerve injuries, which are the most common postoperative complications [5]. Savoca et al. reported on surgical adverse events, adverse medical events, reoperation, and readmission [20]. Surgical adverse events included nerve injuries, wound dehiscence, and surgical site infections, which occurred at rates similar to Drews et al. [20,21]. Medical adverse events omitted hypocalcemia, which was incorporated as a complication by Drews et al. [20,21]. Al-Qurayshi et al. reported surgical and medical complications, including hypocalcemia and nerve injury, and did not find complications that differed by surgical subspecialty [3]. Hypocalcemia, transient or persistent, is the most common complication when reported. The variability in reporting may account for disparities in postoperative hypocalcemia rates, and therefore overall complication rates. The definition of hypocalcemia also varies, making a direct comparison between hypocalcemia rates difficult. Postoperative management differs by surgical subspecialty. Otolaryngologists are more inclined to perform postoperative laryngoscopy and measure serum calcium levels. These differences would falsely elevate postoperative complications in this subspecialty [21]. Numerous studies have compared surgical subspecialty and the association with postoperative outcomes in pediatric thyroidectomy. Unfortunately, the studies are challenging to compare, and provide inconsistent data regarding complications based solely on surgeon subspecialty. Therefore, the one subspecialty cannot be identified as superior at performing pediatric thyroidectomies.

Some difficulty in comparing studies regarding surgical subspecialty arise from the databases utilized and the timeframes. Utria et al. and Savoca et al. used NSQIP-P from different periods [5,20]. This database is data pooled from 100 participating hospitals dedicated explicitly to pediatric care and quality improvement. They may not be fully representative of the breadth of surgeons performing pediatric thyroidectomies. The Pediatric Health Information System (PHIS) was the database used by Drews et al., including 52 free-standing pediatric hospitals [21]. The authors specifically looked at pediatric surgeons only [21]. Al-Qurayshi et al. utilized information from the National Inpatient Sample database, the largest all-payer inpatient database including 1000 hospitals. Still, it includes only inpatient information and does not capture outpatient thyroidectomy procedures [3]. Databases are utilized as representative examples of the general population. However, each database provides different information and includes various participating hospitals. Free-standing children’s hospitals and those voluntarily participating in NSQIP-P tend to be higher-volume, academic, and training facilities that may skew the data from an adequate representation of smaller, community-based hospitals with less surgical sub-specialization patient volume. 

## 3. Impact of Surgeon Volume

While pediatric surgeons perform 75% of thyroidectomies, some data show that pediatric surgeons perform endocrine surgeries on average in 1–2 operations per year [6,8]. Pediatric patients are more likely than adult patients to be treated by a low-volume surgeon [3]. Seventy-four percent of patients are treated by surgeons performing less than 30 thyroid surgeries per year and 27% by surgeons performing two or less thyroid surgeries per year [3]. In one study, 86% of pediatric thyroid cancer patients were treated at low-volume facilities, with the majority treating only one patient per year [6]. Some studies have found pediatric patients most often are treated at teaching hospitals [16]. This highlights the complexity of the definition of a “high-volume pediatric thyroid surgeon”. Pediatric surgeons at teaching hospitals perform most pediatric thyroidectomies, but low-volume surgeons also perform them. 

Complications after adult thyroid surgery are associated with surgeon experience, measured by case volume. Higher volume adult surgeons are defined as performing more than thirty endocrine surgeries per year. They have been shown to have fewer postoperative complications and shorter lengths of stay [18,19]. Complication rates decrease as the case volume increases, with surgeons performing over one hundred endocrine surgeries per year having the lowest complication rates [18,19]. Whether this concept is transferable to pediatric thyroidectomies continues to be debated. 

Postoperative complications following total thyroidectomies in pediatric patients were significantly lower when treated by a high-volume surgeon [3,8,15,21]. Comparison among studies is difficult given the lack of consistency in defining a “high-volume surgeon” and the complications included. Table 3 lists the definitions of high-volume surgeons used for analysis in each study. Tuggle et al. and Sosa et al. found fewer in-hospital complications, shorter length of stays, and lower hospital costs when comparing high-volume surgeons with pediatric surgeons and low-volume general surgeons [8,15]. This suggests that thyroidectomy volume is essential in outcomes regardless of adult or pediatric. Further analysis of surgical volume into low (1–2 surgeries per year), intermediate (3–30 surgeries per year), and high-volume (more than 30 surgeries per year) categories revealed significantly higher complication rates among low volume surgeons [3]. However, similar complication rates among high-volume and intermediate-volume surgeons are reported [3]. This indicates a lower threshold for “high-volume” could be considered in pediatric thyroid surgery. Drews et al. specifically looked at pediatric surgeons only. Surgeon volume ranged from 1–13 surgeries per year, and the top third was considered high-volume (nine or more surgeries per year) [21]. Significantly fewer complications occurred in patients treated by high-volume surgeons [21]. The complication rate reported by Drews et al. for surgeons performing 9–13 surgeries per year was 15%, similar to the 14% complication rate reported by Al-Qurayshi et al. in surgeons performing 3–30 surgeries per year and more than 30 surgeries per year. Bussieres et al. report on a single institution’s experience from 2006–2015. A pediatric surgeon performed approximately ten pediatric thyroid surgeries per year, and the total complication rate was 4.2% [22]. The complications including in these studies differ, making direct comparisons difficult. However, studies suggest higher-volume surgeons, adult or pediatric, are associated with improved outcomes for pediatric thyroid surgery. The threshold for “high-volume” among pediatric thyroid surgery is unclear but is suspected to be lower than the high-volume threshold in adult thyroid surgery. 

The role of high-volume surgeons in higher-risk cases must also be considered. Postoperative complications have been reported as significantly higher in pediatric patients with thyroid malignancy [15,21]. Drews et al. found fewer postoperative complications in patients with malignant disease with a high-volume surgeon (25%) than postoperative complications in patients with malignant disease treated by a low-volume surgeon (35.1%); complication rates for high and low-volume surgeons treating benign disease were similar [21]. Bussieres et al. report a low complication rate, but describe a subset of patients, either very young or with advanced disease, that were more prone to complications; therefore, they recommend similar patients be treated by pediatric general surgeons and pediatric otolaryngologists together [22]. Other studies have also reported a difference in complications by patient age, reporting a higher risk of complications associated with age under six years old [15,16]. Pediatric surgeons are trained in a specific skillset, working in smaller surgical fields and understanding pediatric anatomy transferable to pediatric thyroidectomies. Age and disease pathology in pediatric thyroidectomies has an impact on postoperative complications. In higher risk patients, surgeon volume and pediatric-specific training have an impact on mitigating some risks. Both should be important preoperative considerations. 

In addition to evaluating surgeon volume, hospital volume was a factor considered to affect patient outcomes. Higher hospital volume is hypothesized to correlate with higher volume surgeons and has additional personnel and resources familiar with pediatric thyroid disease. The definition of a high-volume hospital also varied among studies, as highlighted in Table 3. Drews et al. defined a high-volume hospital as those in the top third among their study, correlating 22 or more cases per year. Complication rates were not statistically different based on hospital volume [21]. High-volume hospitals defined as sites with over 51 adult and pediatric thyroidectomies per year did not correlate with decreased postoperative complications [8]. In analyzing hospital volume and 30-day readmission rates, high-volume facilities have calculated an average of three thyroidectomies annually and were associated with lower 30-day readmission rates after pediatric thyroidectomy for malignancy [6]. Cases treated at high-volume facilities were more complex, with higher medullary thyroid cancer and lymphatic spread rates [6]. The hypothesized cause for most readmissions was hypocalcemia [6]. While three pediatric thyroidectomies annually is low compared to the adult numbers, the high-volume facilities in the study were also high-volume adult thyroidectomy facilities. The experience gained from the adult thyroidectomies is suspected to have assisted with the pediatric care [6]. 

Surgeon case volume per year has been associated with decreased complications in adult thyroid surgery. Evidence supports similar conclusions for pediatric thyroidectomies, particularly those performed for malignancy, advanced disease, or children under six years old. However, the case number that constitutes “high-volume” for pediatric thyroidectomies is undefined. 

## 4. Institutional Approaches

Analyses of pediatric thyroidectomies for malignancy reveals the importance of surgeon experience. Given the low incidence of pediatric thyroid malignancy, extensive surgeon experience with pediatric thyroidectomies is challenging to achieve. A common consensus around the importance of a multidisciplinary approach to ensure pediatric-trained and thyroid-experienced physicians are involved in a patient’s care is apparent. This has led to the development of structured multidisciplinary centers dedicated to pediatric patients with thyroid cancer [3,4,6,15]. 

Bussieres et al. discuss their dual-surgeon approach, including a senior and junior pediatric surgeon [22]. Their results support that a lower volume surgeon can have similar outcomes to a high-volume surgeon, and they hypothesize that total lifetime thyroidectomies may be more influential than thyroidectomies per year [22]. Therefore, they created a dual-surgeon approach in which each case involves a senior surgeon and a junior surgeon [22]. The junior surgeon gains experience and case numbers by learning from the senior surgeon [22].

Wood et al. describe their institutional partnership approach between a pediatric surgeon and a high-volume endocrine surgeon [4]. The team works closely with both the children’s hospital and university hospital oncology and radiology departments. Analysis of their outcomes revealed a median length of stay of 1 day, no nerve injuries, and an 11% rate of transient hypocalcemia, similar to other reported complication rates [4]. They conclude that their collaborative surgical approach leads to favorable outcomes [4].

Baumgarten et al. describe a multidisciplinary team (general surgery, otolaryngology, endocrinology, oncology, pathology, radiology, nuclear medicine, social workers, and behavioral health services) at their institution that comprises a Pediatric Thyroid Center [23]. This module has a single contact point for patient families and coordinates patient care at one location [23]. They chose not to have a dual-surgeon approach from a surgical perspective because they are a pediatric surgical training facility [23]. However, this means that those in training are receiving experience with pediatric thyroid surgeries. Surgical outcomes and complication rates, including hypocalcemia and nerve injury, are similar to those reported by other high-volume surgeons, except for a shorter stay [23]. Their case numbers have increased from 23 cases per surgeon in 2011 to over 40 cases per surgeon in 2015 [23]. They do not report a decrease in complication rates with the higher surgical volume [23].

The development of institutional approaches to pediatric thyroid malignancies and high-volume centers for pediatric thyroidectomies has facilitated the care of this patient population. However, access to care remains a concern. Adult surgeons are more likely to treat Caucasian patients, while pediatric surgeons are more likely to treat minorities [5]. Low-volume surgeons more often treat low-income, minority, and uninsured patients [8]. Complication rates, length of stay, and hospital costs are higher for patients from low-income families [15]. High-volume centers are few and far between, adding a logistical and travel cost burden to low-income families. The development of high-volume centers will decrease the number of surgeons willing to perform pediatric thyroidectomies, making access to care more challenging.

## 5. Conclusions

Pediatric thyroid malignancy is a rare disease but is increasing in incidence. Presentation and subtype vary by age, with the most complex cases often presenting in children under six years old. Treatment involves surgical intervention, which depends on the subtype and spread of the disease. Generally, disease-free survival is high, but postoperative complications are not infrequent. Complications are higher in patients with malignancy, more advanced disease, and under six years old.

Pediatric surgeons and low-volume surgeons perform the majority of pediatric thyroidectomies. Adult general surgeons and adult otolaryngologists also contribute to care. Patients under six years old are more likely to be operated on by pediatric surgeons. Patients with malignant disease and those that require a neck dissection are more likely to be operated on by a pediatric otolaryngologist or a non-pediatric physician. This review does not identify one subspecialty as superior at performing pediatric thyroidectomies, as each has unique skills and techniques applicable to pediatric thyroid surgery.

The correlation between high-volume surgeons and lower complication rates in adult thyroid surgery applies to the pediatric population; however, the definition of high-volume for pediatric thyroidectomies requires further investigation. Patients with medullary thyroid cancer, an advanced disease requiring neck dissection, or who are under six years old are at higher risk of complications and benefit from pediatric-trained surgeons and high-volume endocrine surgeons. The development of dedicated pediatric thyroid malignancy centers and multidisciplinary or dual-surgeon approaches are advantageous. Efforts to increase case volume for surgeons by combined or multidisciplinary approaches could increase experience and ultimately improve outcomes.

## Figures and Tables

**Table 1:** Pediatric thyroidectomy complications.

Hypocalcemia (Transient or Permanent)
Hypoparathyroidism (transient or permanent)
Vocal cord immobility (transient or permanent)
Hematoma or bleeding
Hoarseness
Surgical site infection
Wound dehiscence
Readmission

Lists the most common postoperative complications for pediatric thyroid surgery.

**Table 2:** Who is performing pediatric thyroidectomies?

Reference	Database	General Surgeon	Otolaryngologist	Pediatric Otolaryngologist	Pediatric Surgeon
Utria et al. [5]	NSQIP-P 2015–2017	18%	7%	33%	42%
Savoca et al. [20]	NSQIP-P 2012–2016	17.6%	6.1%	35.7%	40.5%
Drews et al. [21]	PHIS 2005–2016			33%	67%
Al-Qurayshi et al. [3]	NIS 2003–2010	81.1%	10.5%		8.4%
Tuggle et al. [8]	NIS 1999–2005	60%	19%	4%	17%

Compares the reported pediatric thyroid surgery rates among different surgical subspecialties.

**Table 3:** What is a high-volume pediatric thyroid surgeon?

Reference	Volume Definitions
Al-Qurayshi et al. [3]	High-volume surgeon: 30 or more adult and pediatric thyroid surgeries per year Intermediate-volume surgeon: 3–30 adult and pediatric thyroid surgeries per year Low-volume surgeon: 1–2 adult and pediatric thyroid surgeries per year
Sosa et al. [15]	High-volume surgeon: 30 or more adult and pediatric thyroid surgeries per year Low-volume surgeon: <30 or more adult and pediatric thyroid surgeries per year
Tuggle et al. [8]	High-volume surgeon: 30 or more adult and pediatric thyroid surgeries per year Low-volume surgeon: <30 adult and pediatric thyroid surgeries per year High-volume facility: 51 or more adult and pediatric thyroid surgeries per year Low-volume facility: <51 adult and pediatric thyroid surgeries per year
Drews et al. [21]	High-volume surgeon: 9 or more pediatric thyroid surgeries in the past 365 days Low-volume surgeon: <9 pediatric thyroid surgeries in the past 365 days High-volume facility: 22 or more pediatric thyroid surgeries in the past 365 days Low-volume facility: <22 pediatric thyroid surgeries in the past 365 days
Youngwirth et al. [6]	High-volume facility: 39 or more pediatric thyroid surgeries over 13 years Low-volume facility: <39 pediatric thyroid surgeries over 13 years

Provides the definitions of high-volume used in the studies reviewed.
